# Standardising robotic system setup: an international expert consensus

**DOI:** 10.1007/s00464-025-12144-y

**Published:** 2025-09-05

**Authors:** T. Shakir, G. Lingam, M. Boal, M. Chand, N. Francis

**Affiliations:** 1 University College London, London, UK; 2 Colorectal Surgery, St Marks Hospital & Academic Institute, London, UK; 3 The Griffin Institute, London, UK

**Keywords:** Robotic surgery, Setup, Docking, Standardisation

## Abstract

**Background:**

Robotic surgery has witnessed rapid growth and development, with a concomitant training requirement. However, educational methods can vary, for example, industry or clinician led. This can result in heterogeneous training. This study aimed to develop a standardised method for the setup and docking of a robotic platform through expert consensus.

**Methods:**

A steering committee of robotic surgeons, surgical care practitioners, and industry representatives formulated initial statements for an online Delphi consensus. The process involved three rounds of voting, with consensus defined as over 80% agreement. Participants included members of societal robotics subcommittees and experienced robotic surgical mentors from 13 countries. The consensus statements were divided into five categories: pre-operative considerations, port placement, driving in and docking, instruments and changes, and undocking and driving out.

**Results:**

57 invitations, alongside a social media advertisement, resulted in 63 responses in round one. Rounds two and three had 54 and 52 responses, respectively (85.7% and 96.3% inter-round response rates). Respondents were from 13 countries, with 73% consultants/attendings, 19% surgical care practitioners/first assistants, and 8% fellows. 73% operate in multiple anatomical regions, primarily pelvic, lower, and upper abdominal. Most respondents had significant robotic experience, with 61.9% involved in over 500 cases. The importance of pre-operative considerations included the necessity of naming components, understanding vision cart functions, and emergency undocking procedures. Standardised port placement and docking techniques were established to ensure optimal access and safety. The use of reverse communication to ensure safety with instrument insertion and changes was emphasised with high levels of consensus.

**Conclusion:**

The consensus identified best practices in hardware knowledge, emergency undocking, port placement, docking, instrument handling, and undocking procedures. This aims to standardise training protocols and has the potential to form part of assessment and accreditation. Further research is required to validate these findings.

**Supplementary Information:**

The online version contains supplementary material available at 10.1007/s00464-025-12144-y.

Robotic surgery has seen rapid growth over the past two decades. Globally, there were over 2.25 million robotic procedures performed in 2023, with overall totals exceeding 14 million [1]. In line with this, a growing number of new robotic systems were installed, with 1370 installations in 2023 [1]. Whilst this provides a platform for surgeons to perform advanced minimally invasive procedures, further training is required for not only for surgeons, but for the entire team, including the bedside assistant. New components, technology, and setup principles are normally taught in a variety of different methods.

Electronic learning and industry-led courses predominate, with a number of surgeon led courses beginning to give a clinically orientated focus [2]. Globally, there are varying training methods. In the USA, there is a standardised robotic training curriculum, with the Fundamentals of Robotic Surgery (FRS) [3]. However, docking represents a relatively limited component when compared with other aspects. In the UK, current advertisements for basic robotic skills or robotic assistant courses reveal three Royal College of Surgeons accredited courses, in addition to 8 separate societal courses. This does not include industry delivered training. Each surgical course may develop its own procedures, which have the potential to lead to inefficiencies with an increased risk of errors. The complexity of robotic systems, combined with the need for precise positioning and calibration, makes standardisation crucial for ensuring safety.

Moreover, the lack of a standardised approach with respect to training is also mirrored with regards to assessment. There currently are no objective means of assessing robotic bedside competence. Achieving accreditation or certification for robotic surgery programmes presents its own set of challenges. One major issue is the lack of uniform standards for accreditation. These varying criteria may make it difficult for institutions to meet all requirements consistently. This can lead to disparities in the quality of robotic surgery programmes across different hospitals and regions.

There is a need to combat heterogeneous training methods and an absence of a uniform standard that may allow for accreditation of patient cart setup. Our study aimed to develop a standardised method for setup and docking of a robotic platform, based on expert consensus opinion.

## Materials and methods

A steering committee was formed to develop initial statements for an online Delphi consensus. This was conducted in three rounds, each open for a period of two weeks for online review and voting. Consensus was defined with a threshold of over 80%.

### Steering committee

The steering group was composed of 2 robotic surgeons [including lead faculty of a societal robotic course for Association of Laparoscopic Surgeons of Great Britain and Ireland (ALSGBI)], 2 robotic surgical care practitioners (with over 1000 case volume), and the lead local industry representative. Industry documentation, in addition to a societal basic skills course manual [4], was reviewed to aid generation of consensus statements.

After initial formulation, independent analysis of statements was performed by each member of the steering group. Final agreement was achieved after in-person review. The statements were transcribed onto an online platform (Google Forms) for electronic distribution.

The steering committee formulated statements which were divided into 5 broad categories: system knowledge, port placement, driving in and docking, instruments and changes, and undocking and driving out. Within each phase further tasks revealed 15 total elements (Supplementary Table 2). A total of 88 statements were included in the first round of the Delphi process.

### Delphi process

The online form was distributed via email to 66 participants in June 2024. Each round was open for a two-week period. Participants were selected due to their presence on societal robotics subcommittees in either a surgeon or nursing role (e.g. SAGES, EAES, ACPGBI) and robotic surgical mentors or proctors. The consensus was also advertised on social media to individuals who had been involved with over 500 cases. Industry representatives were invited to respond; however, they declined. All specialties were included from an international cohort.

Participants were asked to select which statements were essential for those having bedside assistant training. At the conclusion of each section, there was an opportunity to provide free-text feedback on any additional questions that should be included. Questions with 80% or higher consensus were withdrawn before the next round of the survey. Any new statements generated from free text were included in the following round. In subsequent rounds, voters were informed of the results from the previous round, presented as percentages alongside each statement.

## Results

**Fig. 1:**
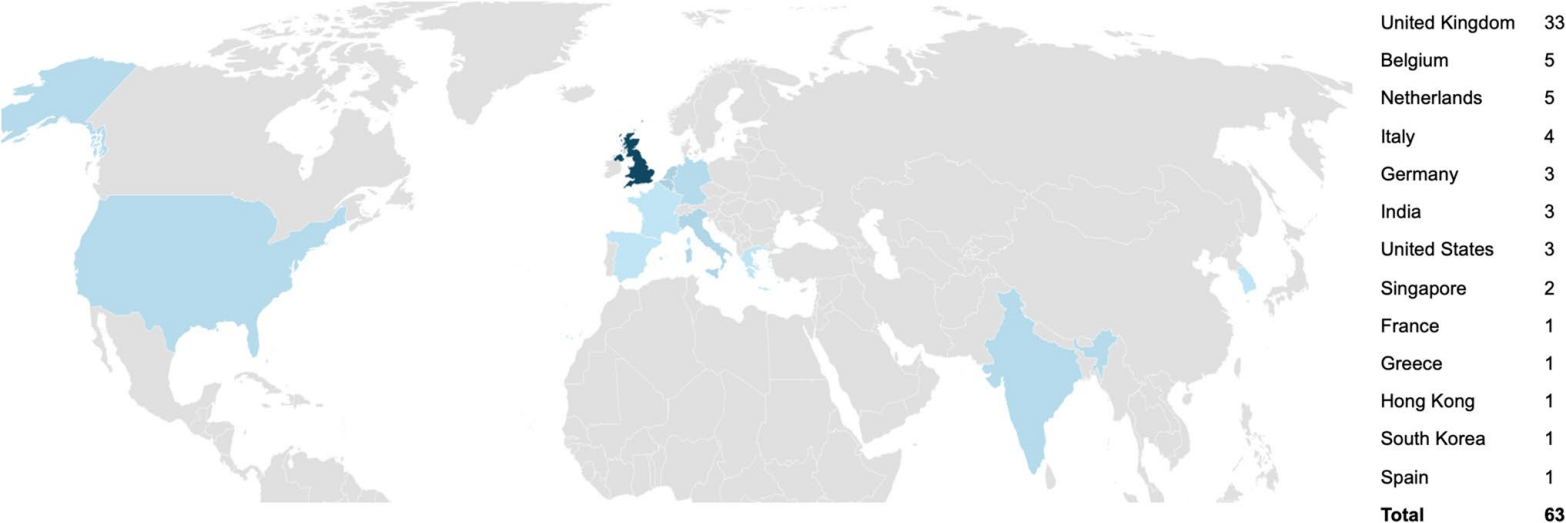
Geographical distribution of responses. Darker shade of blue indicates more responses; grey = no response (Color figure online)

**Fig. 2:**
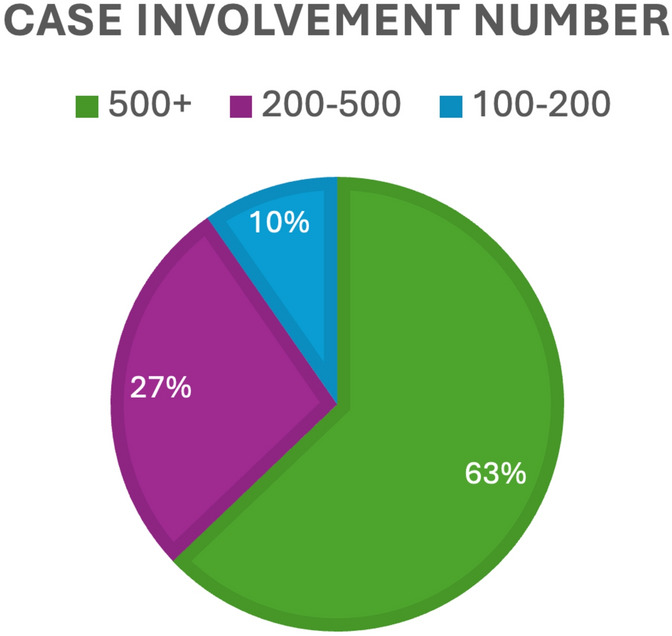
Total number of robotic procedures respondents have been involved with

**Fig. 3:**
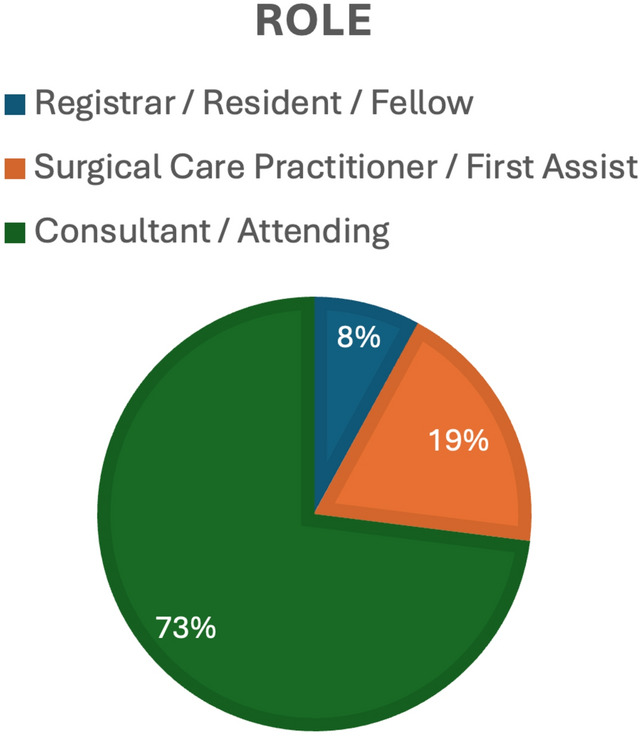
Respondents role within the operating room

57 invitations were sent in round one, in addition to a social media advertisement, with a total of 63 responses received. Due to the advertisement on social media, the true response rate for round one is not known. Respondents were from a total of 13 countries (Fig. 1). 73% were consultant/attending, 19% from surgical care practitioners/first assistants, and 8% from fellows (Fig. 2). Multi-quadrant surgery was highlighted, with 73% of responders indicating they operate in multiple anatomical regions; the three most selected being pelvic, lower, and upper abdominal. The majority of respondents had significant robotic experience; 61.9% had been involved with over 500 cases (Fig. 3).

**Fig. 4:**
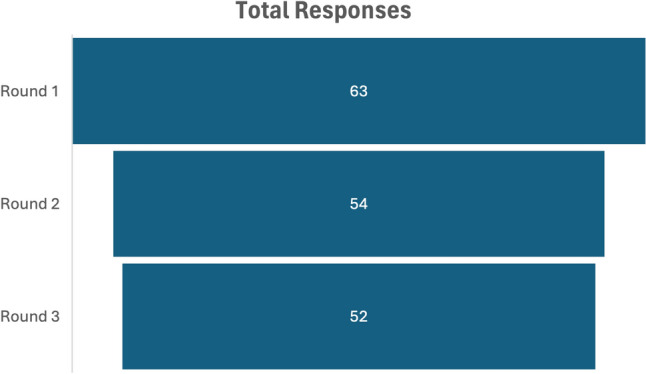
Total number of responses by round

Three further questions were added in the second round based on free-text responses. Round two had 54 responses (85.7% inter-round response rate) and round three concluded with 52 responses (96.3% inter-round response rate) (Fig. 4). Final results are summarised in Table 1, with full results in Supplementary Material.

**Table 1:** Summary of consensus statements

Summary of consensus statements		
Category	Consensus level (High: > 90%, Moderate: 80–90%, None: < 80%)	Findings
*System knowledge*		
Naming components	High	Patient cart, vision cart, surgeon console, instrument clutch, port clutch, remote centre
	Moderate	Energy unit, grab and move, patient clearance buttons, boom rotation button
	None	None
Vision cart functions	High	Pair table
	Moderate	Toggle firefly, toggle camera eye video output
	None	Manage inventory, manipulate patient cart microphone
Endoscope functions	High	Target button functions, orientation changes
	Moderate	Light button, short-press target button
	None	None
Operating table	High	Table motion knowledge, position optimisation
	Moderate	Specialty-dependent pairing
	None	None
Emergency undocking	High	Standardised workflow, emergency key, simulation attendance
	Moderate	Manual lever, power failure/overheating
	None	Roles assignment, conversion tray
*Port placement*	High	Target anatomy identification, port spacing, safety measures, instrument use
	Moderate	Laparoscopy to setup, port hopping, double docking
	None	None
*Driving in and docking*	High	Anatomy selection, cart driving, docking procedures
	Moderate	Cart location, patient side, boom rotation
	None	None
*Instruments and changes*	High	Instrument insertion, control transfer, communication
	Moderate	Instrument changes, LED light colours
	None	30-Degree up camera to facilitate
*Undocking and driving out*	High	Instrument removal, safety checks, sterile stow
	Moderate	Trocar removal under vision, camera light off, driving out
	None	None

For full results, see Supplementary Material

### System knowledge

There was unanimous consensus in round one as to naming all the components (e.g. patient cart, vision cart, surgeon console), in addition to naming buttons (e.g. instrument clutch, port clutch) and endoscope button functionality (e.g. short-press target button to change video output, 30-degree camera manipulations). Participants felt full knowledge of vision cart functions was not required, with two statements not reaching the 80% agreement rate—managing inventory (instrument lives) (56.4%) and manipulating the patient cart microphone (47.3%).

Vision cart functions elicited consensus on three statements: pairing the operating table for mid-case table motion (90.7%), toggling firefly mode on for fluorescence imaging (90.5%), and toggling the video output feed from left to right lens (85.7%). Controlling the patient cart microphone or assessing remaining instrument lives was deemed not necessary. Knowledge of all aspects of endoscope button functionality was deemed required, with high agreement on long-press target button to target (95.2%) and 30-degree camera orientation changes (96.8%).

Key points related to emergency undocking reached consensus. This included knowledge that the patient cart cannot be moved if any port remains docked (92.1%), the importance of attending emergency undocking simulations (95.2%), and the use of the emergency key (93.7%) to manually open instrument jaws. Understanding the manual lever to drive out the robot in case of an emergency was also crucial (84.1%). However, assigning emergency undocking roles during the team brief (47.3%) and a knowledge of what constitutes a standard conversion tray (54.5%) did not achieve consensus.

Participants agreed on the importance of knowledge related to the operating table, with all statements reaching consensus. This included understanding table motion (88.9%), optimising table position to avoid cart clashing when driving in (92.1%), and the dependency of table motion pairing on the surgical specialty (83.6%); for example, thoracic surgery may not be advisable for pairing.

### Port placement

The importance of concepts pertaining to target anatomy and port placement was emphasised, with 100% of statements reaching consensus. Identifying the target anatomy based on the midpoint of the surgical workspace (98.4%) and positioning the endoscope port 10–20 cm from the target (92.1%) were agreed upon. Ports should be placed perpendicular to the target to ensure optimal access and visualisation (82.5%). Knowledge of “double docking”, which involves docking the robot twice during a procedure, was also deemed necessary for certain multi-quadrant or multi-specialty procedures (85.2%). Robotic ports should be spaced 6–8 cm apart, with a range of 4–10 cm (98.4%), and the assistant port should be a minimum of 7 cm away from the robotic ports (90.7%). Marking ports after establishing pneumoperitoneum (85.2%) and avoiding placement within 2 cm of bony landmarks (88.9%) were also highlighted. Performing laparoscopy prior to docking (81.5%) and adjusting port positioning based on anatomical variation (87.3%) were considered moderately important.

### Driving in and docking

Participants emphasised the importance of selecting the correct anatomy and cart location, with over 90% agreement on these points. Deploying for docking (93.7%) and manually manipulating the robot with joysticks (90.5%) had similarly high levels of consensus. Accurate cart driving techniques, such as correctly grasping the handlebars and drive enable switches (96.8%), slowly driving to the operating table whilst monitoring patient clearance (93.7%), and ensuring the correct patient side (88.9%), were emphasised by the majority of participants. The use of reverse communication throughout the process was also widely agreed upon (94.5%).

There was strong consensus on the correct docking of the initial endoscope arm to the initial endoscope port (95.2%), neutralising the horizon (81.0%), and looping the camera cable to avoid tension (82.5%). Subsequent docking procedures, such as docking other arms correctly without error messages (93.7%) and ensuring correct spacing of arms at the boom level (87.3%), were also considered important by the majority of participants. Checking for obstructions when the boom rotates during targeting (88.9%) was another aspect which received high agreement.

### Instruments and changes

Participants reached consensus on the importance of understanding instrument housing LED light colours (90.5%). Safe instrument changes and removal procedures, such as confirming which instruments are to be inserted or removed (96.8%), ensuring instruments are straight (82.5%) and not grasping anything before removal (92.1%), and using guided tool changes (90.5%), were highlighted by the majority. The importance of reverse communication during instrument manipulation was strongly agreed upon (93.7%). In addition, confirming control is handed back to the surgeon console was also agreed upon (90.5%). All elements reached consensus in round one, except for two statements: double-checking remote centre location, which achieved consensus in round three (94.5%), and utilising the 30-degree camera in the up position when visualising instrument insertion, which did not reach consensus (78.2%).

### Undocking and driving out

There was strong agreement on the importance of confirming that all instruments and the camera are removed prior to undocking the port to prevent any damage (95.2%). Turning off the camera light to prevent fire risk was another key safety measure that received moderate consensus (87.0%). Ensuring patient clearance before driving the patient cart away was considered essential by nearly all participants (98.4%). Driving out procedures, including confirming it is safe to proceed with reverse communication (92.1%) and driving the robot away sufficiently prior to sterile stow (87.3%), allowing for space at the bedside, were also widely agreed upon. Sterile stowing procedures were advised to be performed after driving away to ensure sufficient space for the bedside team during closure (92.6%).

## Discussion

Standardisation of techniques pertaining to the setup and docking process of a robotic surgical platform is crucial in line with rapid technological advancements. Current industry-led training programmes are excellent; however, they can lack clinically orientated nuances, especially in the initial stages. The introduction and expansion of clinician-led training courses provide this focus but can be heterogeneous with the influence of anecdotal aspects. These factors, combined with a lack of objective accreditation or certification methods, emphasise a need for a standardised setup protocol. Moreover, this can minimise variability, reduce the risk of errors, and improve overall efficiency [5]. This is especially important in robotic surgery, where precise coordination and operation of new complex equipment are required. Furthermore, often the task of setup and docking is entrusted to an assistant, whereby there is often high turnover, or they may be non-clinical. This therefore underlines the importance of achieving a consensus on the standardised steps for setup.

This study utilised a Delphi approach to ascertain the key components critical to safe and effective setup of the da Vinci Xi platform. This platform was chosen due to its market share and established educational platforms. Although the true response rate from round one was unknown due to social media advertisements, subsequent rounds achieved methodological rigour with responses above the widely accepted guidance of 70% [6]. Overall, 91 statements were evaluated and consensus was achieved on 85 statements to support optimal robotic setup. Five domains were identified, including system knowledge, port placement, driving in and docking, instruments and changes, and undocking and driving out. Although granular statements were generated for each of these sections, the importance of critical aspects should be highlighted.

The majority of respondents were mainly consultants/attendings with high case volume. Importantly, however, almost a quarter of responses were from surgical care practitioners, fellows or equivalent with substantial experience in setting up the robot. A review of the expanding role of the bedside assistant includes main tasks performed to include setup, patient positioning, and docking [7]. Trends towards this are observed with expanding roles of the bedside assistant [8, 9]. Therefore, it could be argued these individuals are better placed to advise on best practices for setup in view of their high case load. Moreover, assistance with multiple specialities adds further insight and experience. Multinational responses add further weight to the generalisability of the consensus statements.

Non-technical and communication skills were emphasised to be paramount. Robotic surgery presents new challenges such as lack of direct vision and excess noise [10], with mechanisms to improve situational awareness fundamental. Throughout each of the domains with respect to team working, the importance of reverse communication was demonstrated. This involves the instruction being read back and confirmed prior to being executed. Moreover, this is critical with regard to instrument changes. A double confirmation of which instrument and which arm instruments are to be removed from was demonstrated as essential by this consensus with > 95% agreement. This would be instructed by the surgeon, with a confirmation by the bedside assistant in an aim to minimise errors. In addition, the critical role played by the bedside assistant was demonstrated with the requirement to visually and verbally verify that instruments were not grasping anything and were straightened. Furthermore, communication also impacts upon driving the patient cart in and out. The avoidance of using directions with “left” and “right” was demonstrated, with consensus achieved on communicating using defined points relative to patient position, such as towards patient head or legs.

Aspects of emergency undocking were explored during the consensus. Key concepts included the presence of a standardised workflow in theatre and attending emergency undocking simulation exercises. A summary reference table for emergency undocking, including controlled and uncontrolled scenarios, is demonstrated in Table 2. Specific points to consider were also highlighted as being the fact that the patient cart cannot be driven away even if one of the robotic arms is docked to a cannula. This may even be the case if the cannula has been removed from the patient whilst the instrument and cannula are still attached in an attempt to rapidly undock. Knowledge of utilisation of manual components was signified. This involves the use of the hexagonal (Allen) key from the sterile set to manually open an instrument tip or the manual drive lever on the patient cart. The manual drive lever is especially important in case of power failure. Interestingly, however, views differed on a number of points. Whilst this may not occur frequently in clinical practice, it is a time-critical procedure. This consensus did not reach the threshold for defining roles during the pre-operative team briefing. This goes against self-published institutional protocols which emphasise delineation of roles [11, 12]. A possible reason may be that it is not thought to be required during every briefing, owing to the rare nature of emergency undocking. This was echoed by the fact that consensus was not reached on the statement introduced in round 2 involving knowledge of what constitutes a conversion tray.

**Table 2:** Quick reference guide for emergency undocking and troubleshooting

	Controlled emergency undock	Uncontrolled emergency undock
Situation	Bleeding controlled with manual pressure of robotic arm	Bleeding unable to be controlled robotically Cardiorespiratory compromise
Consider	Robot cannot be driven away if one arm remains docked Lateral arm (ideally ipsilateral side to patient cart) to be used for control of bleeding to allow space for surgical team	Prepare sterile tray for conversion Use two hands to remove two instruments at one time Additional help
Roles and responsibilities	1. Surgeon to alert and scrub 2. Scrub nurse to prepare sterile tray for conversion 3. Circulator to drive robot away monitoring for clearance 4. First assistant to remove instruments and undock. Care to not remove instrument controlling bleeding	1–3: As controlled undock *If first assistant and scrub nurse on ****same**** side:* First assistant to remove instruments and undock two arms on *opposite* side Scrub nurse to remove instruments and undock two arms on *same* side *If first assistant and scrub nurse on ****opposite**** sides:* First assistant and scrub nurse to remove 2 instruments and undock arms on respective sides
Troubleshooting	Faults *Recoverable*—Orange lights—Can press “recover fault” button on surgeon console touchpad, patient cart touchpad or vision cart screen *Non-recoverable*—Red lights—Power cycle to restore error. Not required to remove instruments / endoscope Use of emergency key in case of instrument failure Locate manual instrument release kit within sterile tray Insert Allen key into grip release section of instrument housing Turn in direction of arrow Note instrument should not be re-used after using the instrument release tool Use of manual drive if power loss *NB: Ensure robot is not docked to patient* Open access panel on front of patient cart base Turn red lever to enable manual cart driving Two people to move cart due to weight (840 kg) Return red lever to original position (powered)	

Refs: Da Vinci Xi Surgical System In-Service Guide: OR Staff; OR Set-Up Guide Clinical Specialty Guide. Intuitive Surgical

**Fig. 5:**
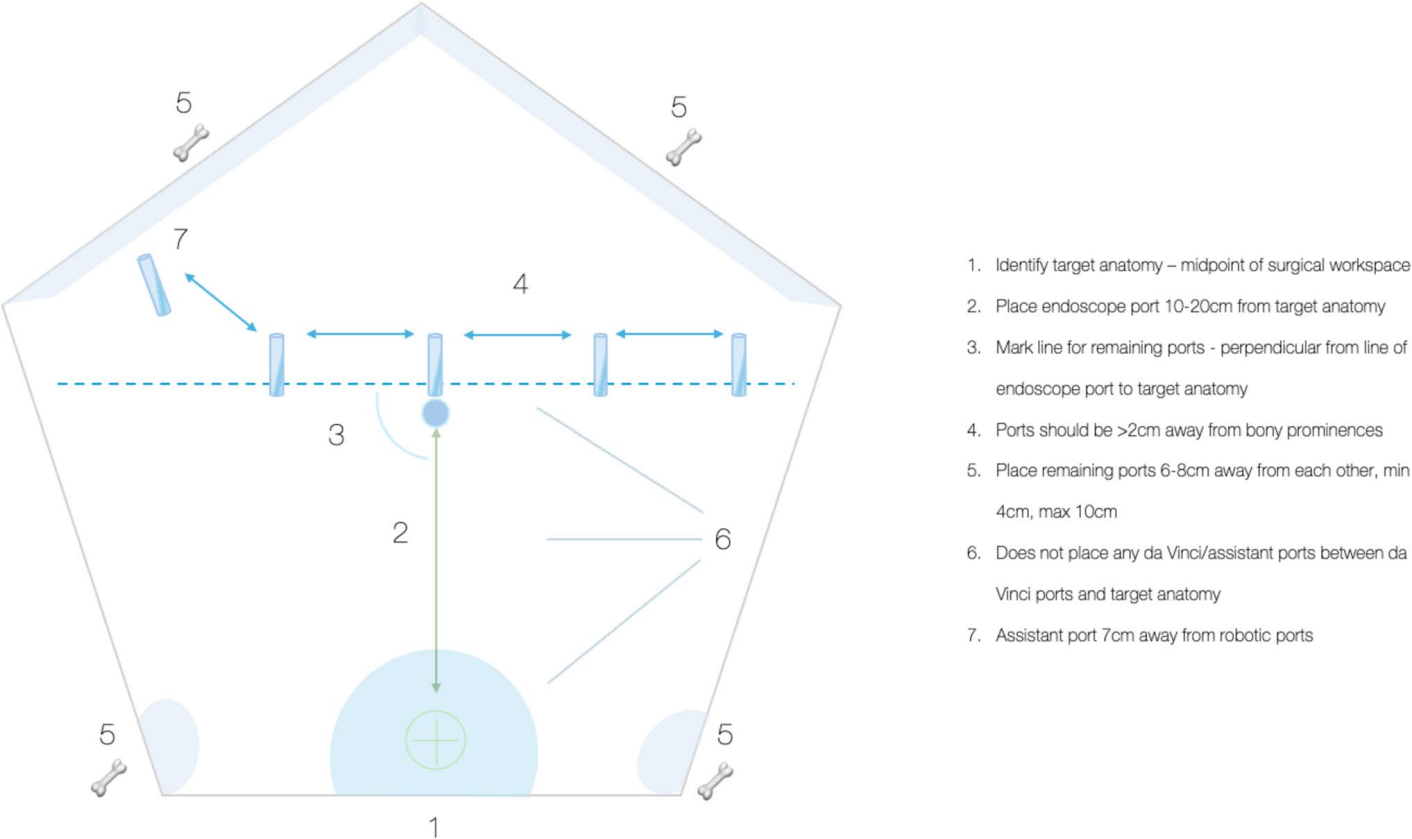
Summary of key principles of port placement

Aspects pertaining to port placement all reached consensus. This is likely consequent due to the rigid nature of port placement within this particular system. Boom-mounted robotic systems have certain hardware-based constraints with regard to arm clashing. As such, industry-defined specifications are required to be followed to ensure usability. Although some deviation with port placement is permissible, the overarching principles remain, with optimal port placement strategies being demonstrated by consensus statements. A summary of key points regarding port placement is highlighted in Fig. 5. These principles, if followed, would allow for optimal multi-quadrant use; however, the concept of double docking would further this. This is the process of, once undocked, rotating the boom to the opposite direction to enable operating in an opposite quadrant. This can be enabled from the patient cart touchpad or manually through the use of the boom rotation buttons on arms 1 and 4. Especially pertinent to facilitate multi-field procedures is the use of integrated table motion. This allows for the table position to be altered even when the robot is docked. This is enabled through pairing the specific patient table with the robot through a wireless connection (Table 3).

**Table 3:** Guide for use of integrated table motion

Table motion	
*How to pair*	
Press dV button on upper middle of table remote control (NB: requires procurement of specific table)	
Press Pair function on vision cart touchscreen (NB have limited time to perform this before exits pairing mode)	
Confirm pairing with visual and audio feedback	
*How to move table when docked*	
Remove 1 instrument (from side with 2 instruments) so surgeon in full control of all instruments at once from console	
Anaesthesiologist to press dV button on remote	
Audio feedback to confirm ready for table motion	
Surgeon to remain in control of instruments from within console	
Anaesthesiologist to move table to desired position	

## Limitations

Despite the majority of topics reaching consensus, there are limitations with this Delphi approach. Initial statement formulation is subject to bias, with the initial steering committee formulating the statements. This is attempted to be mitigated by the option of a free-text response to add additional statements. Nonetheless, this would rely on motivated individuals completing the consensus to critically analyse the listed statements and formulate new ones based on knowledge gaps identified. Furthermore, expert opinion represents a low level of evidence and should be factored appropriately. The utilisation of these consensus statements may be multifactorial. This could form part of standardised training curricula or be utilised in assessment or accreditation. However, there is a requirement to validate this prior to use in any setting, forming part of further work. Additionally, by virtue of differing hardware components, the nature of this consensus is platform specific. With a drive towards platform agnosticism, this represents a limitation and this work should be expanded to multiple platforms.

## Conclusion

Best practice was identified with respect to hardware knowledge, emergency undocking, port placement, docking and instruments, and undocking and driving out. This consensus aims to standardise training, with further research required to validate these findings.

## Supplementary Information

Below is the link to the electronic supplementary material.

Supplementary file1 (DOCX 17 KB)

Supplementary file2 (DOCX 29 KB)
