# Supplementary material for: Standardising robotic system setup: an international expert consensus
Source: Surg Endosc. 2025 Sep 5;39(11):7640–8. doi: 10.1007/s00464-025-12144-y (PMC12618431; doi:10.1007/s00464-025-12144-y)
Supplement: Supplementary file 1 — Supplementary file1 (DOCX 17 KB) [file 464_2025_12144_MOESM1_ESM.docx]

| Total Responses |  |  |
| --- | --- | --- |
| Round 1 | 63 |  |
| Round 2 | 54 | 85.7% IRR |
| Round 3 | 52 | 96.3% IRR |
|  |  |  |
| Anatomical Region (Multiple Options Allowed) |  |  |
| Pelvic | 39 |  |
| Lower Abdominal | 36 |  |
| Upper Abdominal | 18 |  |
| Renal | 10 |  |
| Thoracic | 7 |  |
| Transoral | 2 |  |
| Abdominal Wall | 1 |  |
|  |  |  |
| Country |  |  |
| United Kingdom | 33 | 52.38% |
| Belgium | 5 | 7.94% |
| Netherlands | 5 | 7.94% |
| Italy | 4 | 6.35% |
| Germany | 3 | 4.76% |
| India | 3 | 4.76% |
| United States | 2 | 4.76% |
| Singapore | 2 | 3.17% |
| South Korea | 1 | 1.59% |
| Greece | 1 | 1.59% |
| France | 1 | 1.59% |
| Spain | 1 | 1.59% |
| Hong Kong | 1 | 1.59% |
|  |  |  |
| Case Involvement Number |  |  |
| 500+ | 39 | 61.90% |
| 200-500 | 17 | 26.98% |
| 100-200 | 6 | 9.52% |
|  |  |  |
| Role |  |  |
| Registrar / Resident / Fellow | 5 | 7.94% |
| Surgical Care Practitioner / First Assist | 12 | 19.05% |
| Consultant / Attending | 46 | 73.02% |
