# Supplementary material for: Standardising robotic system setup: an international expert consensus
Source: Surg Endosc. 2025 Sep 5;39(11):7640–8. doi: 10.1007/s00464-025-12144-y (PMC12618431; doi:10.1007/s00464-025-12144-y)
Supplement: Supplementary file 2 — Supplementary file2 (DOCX 29 KB) [file 464_2025_12144_MOESM2_ESM.docx]

| **Element** | **Yes** | **No** | **Round Achieved Consensus** | |
| --- | --- | --- | --- | --- |
| System Knowledge |  |  | |  |
| **Naming Components / Buttonology** |  |  | |  |
| Patient Cart | 98.40% | 1.60% | | 1 |
| Vision Cart | 98.40% | 1.60% | | 1 |
| Surgeon Console | 98.40% | 1.60% | | 1 |
| Energy unit | 90.50% | 9.50% | | 1 |
| Instrument clutch | 98.40% | 1.60% | | 1 |
| Port clutch | 98.40% | 1.60% | | 1 |
| Grab and move | 84.40% | 15.60% | | 1 |
| Patient clearance buttons | 93.70% | 6.30% | | 1 |
| Boom rotation button | 87.30% | 12.70% | | 1 |
| Remote Centre | 98.40% | 1.60% | | 1 |
|  |  |  | |  |
| **Vision Cart Functions** |  |  | |  |
| Pair table | 90.70% | 9.30% | | 2 |
| Manage inventory (i.e. instrument lives) | 56.40% | 43.60% | | N/A |
| Toggle firefly (ICG mode) | 90.50% | 9.50% | | 1 |
| Toggle camera eye video output | 85.70% | 14.30% | | 1 |
| Manipulate patient cart microphone | 47.30% | 52.70% | | N/A |
|  |  |  | |  |
| **Endoscope Functions** |  |  | |  |
| Long press target button to target | 95.20% | 4.80% | | 1 |
| Short press target button to change eye video output | 88.90% | 11.10% | | 1 |
| Long press light button to toggle endoscope light | 85.70% | 14.30% | | 1 |
| 30up and 30down orientation changes | 96.80% | 3.20% | | 1 |
|  |  |  | |  |
| **Emergency Undocking** |  |  | |  |
| Roles to be assigned during team brief | 47.30% | 52.70% | | N/A |
| Standardised workflow in theatre | 90.70% | 9.30% | | 2 |
| Knowledge that cannot move robot if port still docked | 92.10% | 7.90% | | 1 |
| Should attend emergency undocking simulation | 95.20% | 4.80% | | 1 |
| How to use emergency key | 93.70% | 6.30% | | 1 |
| Knowledge of manual lever to drive out | 84.10% | 15.90% | | 1 |
| NEW Q - Know what to do in case of power failure / overheating | 89.10% | 10.90% | | 3 |
| NEW Q - what constitutes a standard conversion tray | 54.50% | 45.50% | | N/A |
|  |  |  | |  |
| **Operating table** |  |  | |  |
| Knowledge of table motion (if applicable) | 88.90% | 11.10% | | 1 |
| Optimises table position to avoid robot clashing | 92.10% | 7.90% | | 1 |
| Table motion pairing dependent on speciality (i.e. thoracic not advisable for pairing) | 83.60% | 16.40% | | 3 |
|  |  |  | |  |
| Port Placement |  |  | |  |
| **Target anatomy:** |  |  | |  |
| Identifies target anatomy based on operation (midpoint of surgical workspace) | 98.40% | 1.60% | | 1 |
| Endoscope port 10-20cm from target | 92.10% | 7.90% | | 1 |
| Ports placed perpendicular to target | 82.50% | 17.50% | | 1 |
| NEW Q - how to "double dock" | 85.20% | 14.80% | | 2 |
|  |  |  | |  |
| **Ports:** |  |  | |  |
| Places ports 6-8cm apart (range 4 - 10cm) | 98.40% | 1.60% | | 1 |
| Assistant port minimum 7cm away from robotic ports | 90.70% | 9.30% | | 2 |
| Marks ports after establishing pneumoperitoneum | 85.20% | 14.80% | | 2 |
| Does not place ports less than 2cm from bony landmarks | 88.90% | 11.10% | | 1 |
| Does not place any da Vinci/assistant ports between da Vinci ports and target anatomy | 88.90% | 11.10% | | 2 |
| Considers performing laparoscopy prior to docking for setup | 81.50% | 18.50% | | 2 |
| Able to insert / upsize 12mm port safely | 92.10% | 7.90% | | 1 |
| Correctly uses reducer when using 8mm instrument in 12mm port | 98.40% | 1.60% | | 1 |
| Knowledge to adjust port positioning based on anatomic variation | 87.30% | 12.70% | | 1 |
| Knowledge can use only 3 arms | 87.30% | 12.70% | | 3 |
| Knowledge of port hopping (e.g. when 5 robotic ports, or swapping camera port) | 90.50% | 9.50% | | 1 |
|  |  |  | |  |
| Driving in and Docking |  |  | |  |
| **From cart touchpad** |  |  | |  |
| Selects correct anatomy (upper abdomen, pelvic, thoracic etc) | 92.10% | 7.90% | | 1 |
| Selects correct cart location | 88.90% | 11.10% | | 1 |
| Presses and holds deploy for docking until completion | 93.70% | 6.30% | | 1 |
| Able to manually manipulate robot with joysticks | 90.50% | 9.50% | | 1 |
|  |  |  | |  |
| **Cart driving:** |  |  | |  |
| Correctly grasps handlebars and drive enable switches | 96.80% | 3.20% | | 1 |
| Slowly drives to operating table monitoring patient clearance | 93.70% | 6.30% | | 1 |
| Ensures correct patient side as defined during deploy for docking | 88.90% | 11.10% | | 1 |
| Drives laser line within 5cm of initial endoscope port | 90.50% | 9.50% | | 1 |
| Avoids boom rotated 180degrees opposite to base | 83.30% | 16.70% | | 2 |
| Uses reverse communication throughout with defined points of reference relative to patient position | 94.50% | 5.50% | | 3 |
|  |  |  | |  |
| **Docking / Instruments** |  |  | |  |
| Docks initial endoscope arm to initial endoscope port correctly | 95.20% | 4.80% | | 1 |
| Neutralises horizon | 81.00% | 19.00% | | 1 |
| Loops camera cable over endoscope to avoid hanging and tension | 82.50% | 17.50% | | 1 |
| Correctly long presses on target button at target anatomy | 93.70% | 6.30% | | 1 |
| Holds cannula to support during motion | 85.70% | 14.30% | | 1 |
| Ensures endoscope arm is parallel to blue vertical crosshair line on boom (or equivalent) | 96.30% | 3.70% | | 2 |
| Checks for obstructions when boom rotates during targeting | 88.90% | 11.10% | | 1 |
|  |  |  | |  |
| **Subsequent docking** |  |  | |  |
| Docks other arms correctly with no error message/sound | 93.70% | 6.30% | | 1 |
| Docks medial arm first for ease (e.g. 3 prior to 4) | 40.00% | 60.00% | | N/A |
| Checks remote centres | 84.10% | 15.90% | | 1 |
| Uses camera from a different port to check remote centre of initial endoscope port | 87.30% | 12.70% | | 3 |
| Burps ports to release tension on abdominal wall | 92.10% | 7.90% | | 1 |
| Ensures hand breadth between arms | 92.10% | 7.90% | | 1 |
| Ensures correct spacing of arms at boom level | 87.30% | 12.70% | | 1 |
|  |  |  | |  |
| Instruments and Changes |  |  | |  |
| **Instrument insertion** |  |  | |  |
| Confirms which instruments to be inserted into which arms with reverse communication | 93.70% | 6.30% | | 1 |
| Inserts instrument under direct vision from trocar | 92.10% | 7.90% | | 1 |
| Double checks remote centre location | 94.50% | 5.50% | | 3 |
| Utilises 30 degree up camera if available | 78.20% | 21.80% | | N/A |
| Correctly gives control of all instruments to surgeon | 93.70% | 6.30% | | 1 |
| Correctly gives control of camera to surgeon | 93.70% | 6.30% | | 1 |
| Confirms diathermy connected if applicable and appropriately placed with reverse communication | 88.90% | 11.10% | | 1 |
| Confirms robot at control of surgeon console with reverse communication | 85.70% | 14.30% | | 1 |
|  |  |  | |  |
| **Instrument changes / removal** |  |  | |  |
| Uses reverse communication for all applicable steps | 95.20% | 4.80% | | 1 |
| Confirms which instrument to be removed from which arm | 96.80% | 3.20% | | 1 |
| Confirms instrument not grasping anything | 92.10% | 7.90% | | 1 |
| Confirms instrument wrist straightened | 82.50% | 17.50% | | 1 |
| Inserts new instrument with guided tool change (green light) | 90.50% | 9.50% | | 1 |
| Confirms instrument inserted fully and control back to surgeon | 90.50% | 9.50% | | 1 |
| Understands instrument housing LED lights colours (e.g. flashing blue = control at patient cart) | 90.50% | 9.50% | | 1 |
|  |  |  | |  |
| Undocking and Driving Out |  |  | |  |
| **Undocking** |  |  | |  |
| Confirms instruments and camera are removed prior to undocking port | 95.20% | 4.80% | | 1 |
| Removes trocars under vision to check for bleeding | 84.10% | 15.90% | | 1 |
| Turns off camera light to prevent fire risk | 87.00% | 13.00% | | 2 |
| Confirms patient clear prior to robot being driven away | 98.40% | 1.60% | | 1 |
| Instructs for robot to be driven away | 85.70% | 14.30% | | 1 |
|  |  |  | |  |
| **Driving out** |  |  | |  |
| Confirms safe to proceed with reverse communication | 92.10% | 7.90% | | 1 |
| Drives robot away correctly prior to sterile stow | 87.30% | 12.70% | | 1 |
| Sterile stows correctly | 92.60% | 7.40% | | 2 |
